# Effect of Protonated Media on Dye Diffusion in Chitosan–Cellulose-Based Cryogel Beads

**DOI:** 10.3390/gels11100770

**Published:** 2025-09-25

**Authors:** Alfredo García-González, Rosa Elvira Zavala-Arce, Pedro Avila-Pérez, Jacob Josafat Salazar-Rábago, Jose Luis Garcia-Rivas, Carlos Eduardo Barrera-Díaz

**Affiliations:** 1 Tecnológico Nacional de México/Instituto Tecnológico de Toluca, Av. Tecnológico s/n. Colonia Agrícola Bellavista, Metepec C.P. 52149, Estado de México, Mexico; alfredo.gc@toluca.tecnm.mx (A.G.-G.); jgarciar@toluca.tecnm.mx (J.L.G.-R.); 2 Centro Conjunto de Investigación en Química Sustentable UAEM—UNAM, Carretera Toluca-Atlacomulco Km 14.5, Unidad El Rosedal, Toluca C.P. 50200, Estado de México, Mexico; pavilap@uaemex.mx (P.A.-P.); cebarrerad@uaemex.mx (C.E.B.-D.); 3 Facultad de Ciencias Químicas, Universidad Autónoma de Nuevo León, Ave. Universidad S/N Cd. Universitaria, San Nicolás de los Garza C.P. 66455, Nuevo León, Mexico; jacob.salazarrb@uanl.edu.mx

**Keywords:** chitosan–cellulose, protoned, geometry, adsorption, anionic dye, cryogel

## Abstract

Synthetic dyes are increasingly relevant pollutants due to their widespread use and discharge into water bodies. This study examines how the solution pH affects the morphology of chitosan–cellulose cryogel (Ch-C-EGDE) and its impact on dye transport to adsorption sites. Adsorption tests with dyes Y5, R2, and B1 over a pH range of 2–12 revealed optimal performance at pH 2.5. High hydronium ion concentrations significantly improved adsorption capacities (945–1605 mg/g), with a hierarchy B1 > R2 > Y5 at 250 mg/L initial concentration. The dependence of the dye adsorption on the acidic pH of the solution suggests that there is a mechanism of adsorption by electrostatic forces due mainly to the protonation of the amino group (NH_3_^+^). During the dye adsorption studies, a decrease in the diameter of the cryogel beads was observed, as well as a possible “zipper effect” in the pores of the Ch-C-EGDE cryogel beads, which depends on the pH at which the anionic molecules of the dyes attract the positively charged chitosan-based adsorbent walls, which physically closes the pores and results in a decrease in pore size as well as a geometric and/or load-bearing impediment. The experimental data fitted well with the pseudo-second-order kinetic models and the Sips isotherm model, indicating multilayer and heterogeneous adsorption behavior. In the Sips model, a value of n > 1 was obtained, which confirms favorable adsorption conditions and suggests strong dye-adsorbent material interactions, especially at higher dye concentrations.

## 1. Introduction

Synthetic dyes are widely used in the food and textile industries, where they often cannot be removed during conventional wastewater treatment. However, even trace amounts of these recalcitrant pollutants can persist in surface waters, reducing light penetration, inhibiting photosynthesis, and ultimately harming aquatic life [1,2].

Various dye removal techniques have been considered, including chemical oxidation, photodegradation, membrane filtration, and biological treatment. Each method presents specific advantages and limitations; for instance, chemical oxidation may involve high operational costs and the generation of harmful byproducts, while biological treatment often exhibits low efficiency in removing recalcitrant dyes. In contrast, adsorption stands out for its operational simplicity, economic viability, high removal efficiency, and the potential for adsorbent regeneration, positioning it as a particularly promising strategy for the remediation of dye-laden wastewater [3].

In order to overcome these drawbacks, adsorption represents an economical and efficient option for dye removal. In this process, solute molecules accumulate on the adsorbent surface due to specific affinities. Among the various adsorbent materials as an alternative for the removal of water color, zeolites, hydrotalcites, activated carbon, and chitosan-based polymeric materials stand out [4,5,6,7,8].

Among the many adsorbents available, the biopolymer chitosan stands out because it is abundant, cost-effective, and easy to process. Furthermore, the structure contains amino and hydroxyl groups that confer a strong affinity for anionic dyes. These properties make chitosan a strong candidate for sustainable and efficient wastewater treatment applications [9].

Chitosan is a polysaccharide derived from the partial deacetylation of chitin, primarily sourced from the exoskeletons of crustaceans, such as shrimp, making it widely available worldwide. Its chemical structure comprises functional groups such as -NH_2_, -COOH, and -OH, which, when dissociated, enable interactions with various elements in solution, including dyes, ions, and dissolved metals [9,10,11,12].

Building on its versatile chemistry, chitosan has been modified and used in different forms—coagulants, flocculants, and composite biosorbents—to enhance its adsorption performance [13,14,15,16,17,18]. Among these applications, cryogelation offers a unique pathway: by freezing polymer solutions, macroporous networks (“cryogels”) are obtained that combine high surface area with structural robustness [19,20,21,22,23].

During the synthesis stage of certain sorbents, various parameters have been found to affect swelling, such as cross-linking dose, pore size, and polymer heterogeneity. In chitosan-based materials, in addition to the characteristics mentioned above, the interaction between the functional groups of the material and the components of the liquid medium, such as dyes, can also affect swelling and, consequently, the diffusion mechanisms of the dyes within the biosorbents [4,10,13,24,25].

Several studies have shown that the amino-NH_2_ group reacts readily in acidic media with HO_3_^+^ hydronium ions, resulting in its protonation (NH_3_^+^) and acquisition of a positive charge. This allows it to interact with other elements in solution [9,11,26,27,28,29].

Chitosan–cellulose cryogels combine high porosity and mechanical strength, making them excellent adsorbents [28,30,31,32,33]. During pH-dependent adsorption tests, cryogel beads showed notable reversible diameter changes, a phenomenon called ‘zipper effect’, which is the basis for their unique dye-diffusion behavior. The main objective of this study is to investigate the effect of ions and solution pH on the morphology of chitosan-based cryogel beads, which, when modified, affects the adsorption process of the dyes under kinetic and equilibrium conditions.

## 2. Results and Discussion

**Figure 1:**
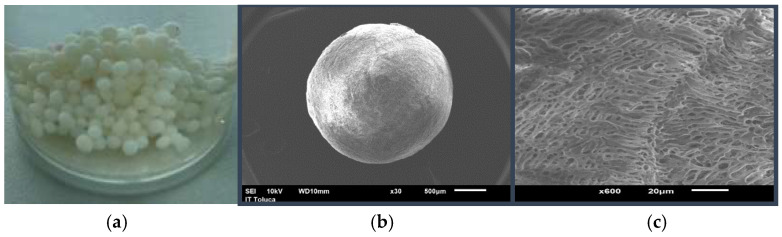
(**a**) Synthesized Ch-C-EGDE cryogel beads in water, (**b**) SEM micrograph of cryogel beads at 30×, (**c**) SEM micrograph of cryogel beads at 600×.

Figure 1a shows an image of the synthesized Ch-C-EGDE cryogel beads, which appear smooth but resist deformation under compression. After hydration, the beads become more sensitive to localized pressure but retain structural stability under agitation. Figure 1b and Figure 1c present SEM micrographs of freeze-dried beads at 30× and 600× magnifications, respectively. These images confirm a uniform spherical morphology with an extensive network of interconnected pores. Cellulose is homogeneously incorporated into the pore walls, reinforcing the beads’ rigidity and mechanical strength [34].

Figure 1c shows that cryogels are formed homogeneously with large porous networks, featuring a pore size ranging from 4 to 5 μm. In addition, in the cuts made to the material, the formation of channels that converge in the center of the beads due to the radial freezing front has been confirmed [19,21,35].

The geometric conformation of the N_2_ adsorption isotherms turned out to be typical of mesoporous adsorbents, where the walls are composed of macroporous networks that are not visible through MEB analysis [11,36]. Table 1 shows the specific surface area results obtained by fitting the N_2_ physisorption data to the BET equation for cryogel beads.

The specific surface area of the synthesized cryogel was 10.22 m^2^/g, which is lower than previously reported values for pure chitosan cryogels (36.67 m^2^/g) and chitosan–cellulose composites (16 m^2^/g); however, this value is within the same order of magnitude for chitosan-based adsorbents [35].

### 2.1. pH Point of Zero Charge Results

**Figure 2:**
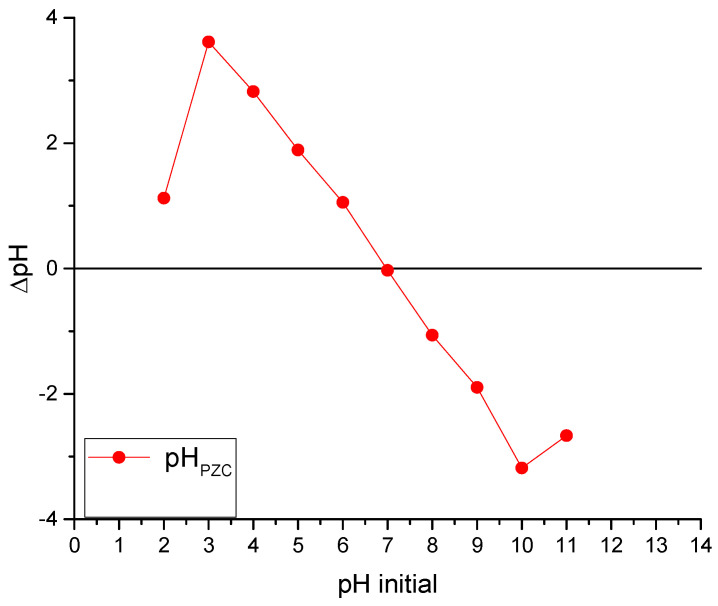
Determination of the point of zero charge (PZC) in the cryogel Ch-C-EGDE.

Figure 2 shows the results of the point of zero charge test applied to chitosan-based Ch-C-EGDE material, which allows the surface charge of the material to be established at different pH values.

The adsorbent surface with a point of zero charge (pHpzc) of 6.7, in a solution with pH below this value, will be predominantly positively charged due to protonation of amino groups present on the chitosan surface. In this acidic range (pH < 6.7), the cationic adsorbent exhibits high affinity for anionic dyes due to electrostatic attraction between negatively charged dye molecules and positively charged adsorption sites. This behavior is consistent with previous studies on chitosan-based materials, which report pHpzc values ranging from 6.3 to 7.2 depending on the degree of deacetylation and structural modifications [37,38]. For instance, Díaz-Flores et al. (2024) observed similar adsorption trends for methylene blue on chitosan–zeolite composites with a pHpzc close to 6.5 [39]. Therefore, under acidic conditions, the adsorbent is highly effective in removing anionic species from solution, as confirmed by both experimental data and literature precedent.

**Figure 3:**
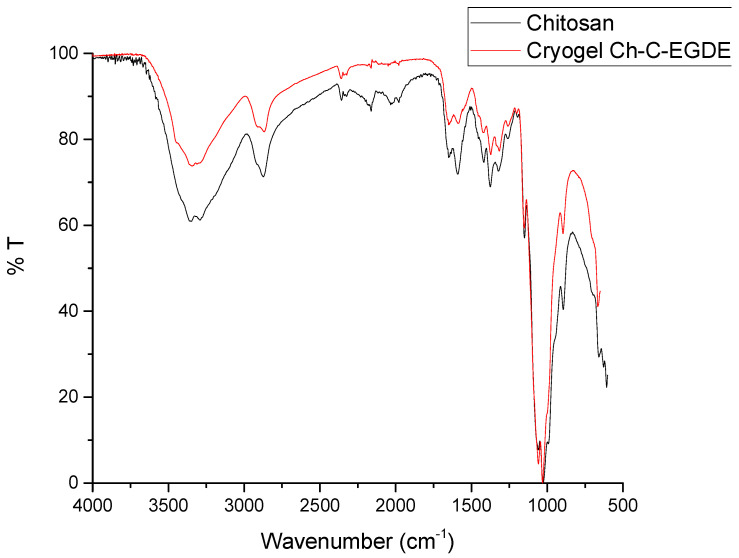
Spectrum of cryogel Ch-C-EGDE via FTIR infrared spectroscopy.

Figure 3 shows the FTIR spectrum of lyophilized Ch-C-EGDE cryogenic beads. The bands at 1635 and 1520 cm^−1^ indicate an asymmetric stretching of the N-H and C-N bonds, respectively. This is corroborated by the stretch at 3300 cm^−1^, which corresponds to the amino group of chitosan [40]. These results support the presence of amino groups during the synthesis of chitosan-based cryogel, which are available for protonation and interaction with dyes in solution.

**Figure 4:**
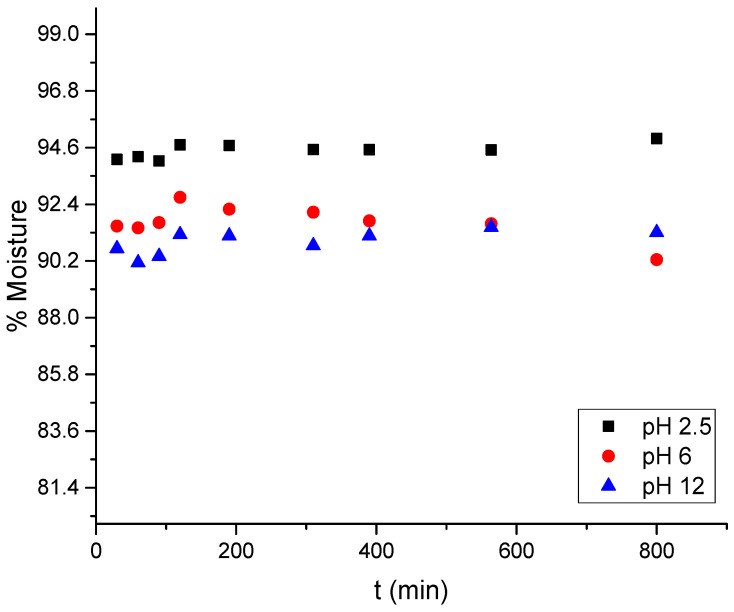
Swelling behavior of cryogel beads at pH 2.5, 6, and 12.

The biosorbents generated are hydrophilic, which is related to the water absorbed in the pores of the cryogel [4,41,42]. The cryogel reached a swelling kinetic equilibrium after 4 h, with a moisture percentage of 90.25 ± 0.46% at a neutral pH (Figure 4). At pH 2.5, the swelling kinetics reached kinetic equilibrium in 1 h, with 94.94 ± 0.13% moisture, while at pH 12, equilibrium was achieved after 2 h, with 91.30% moisture. Bead diameters were measured under neutral (pH 6.0), acidic (pH 2.5), and alkaline (pH 12.0) conditions to quantify pH-induced morphological changes. At pH 6.0, the beads exhibited an average diameter of 2.54 ± 0.30 mm. At pH 2.5, they expanded to 4.57 ± 0.25 mm, an 80% increase, whereas at pH 12.0, they contracted to 1.02 ± 0.15 mm, a 60% reduction. These reversible dimensional changes corroborate the protonation-driven “zipper effect”, which directly modulates dye diffusion and active-site accessibility. At a pH of 2.5, increasing moisture percentage causes the cryogel beads to expand in diameter; however, during the solubility determination tests, it was observed that there is a weight loss of the cryogel beads due to dissolution or loss of constituent elements, which is minimal, <0.01 wt.%. Conversely, at a pH of 12, increasing moisture percentage causes the diameter of the cryogenic beads to decrease proportionally. This suggests a change in the geometry of the bead and its pores in relation to pH [43].

Figure 4 shows the rapid wetting of the material upon contact with various solutions. However, the equilibrium times differ at pH 2.5 and 6–12, being 30 and 60 min, respectively. This kinetic equilibrium is due to the interaction between the functional groups of chitosan and the H^+^ and OH^−^ groups of the solution, a process known as protonation and deprotonation [9,11,12].

Based on previous experiments, the optimal pH of 2.5 was selected, which achieves the highest adsorption capacity. The experimental data were adjusted to the PFO and PSO adsorption models, considering that they can be applicable because they consider that a control step is the rate of molecular diffusion in the pores [44].

The results shown in Figure 4 and the kinetic constants of Ho’s model (PSO) shown in Table 2, allow comparing the rate at which hydration of the material occurs at different pH values and which are related to protonation and deprotonation in acidic and alkaline media. The value obtained of K_2_ = 5.26 at pH 2.5 is higher than K_2_ = 3.786 at pH 12; therefore, it is possible to affirm that protonation is a fast process compared to deprotonation, it is also observed that these processes modify the water retention capacity of the chitosan-based cryogel, where the empirical model assumes a relationship between the rate of decrease in vacant active sites in an infinite medium, as appropriate for water absorption [45].

The effect of the acidic medium on the morphology of the cryogel pore is primarily linked to the amino groups of chitosan. This work identified the presence of these groups through FTIR analysis. The amino groups react with the hydronium ions in the solution, as shown in the following reaction [11,46]:

Q-NH_2_ + H^+^ → Q-NH_3_ (1)

**Figure 5:**
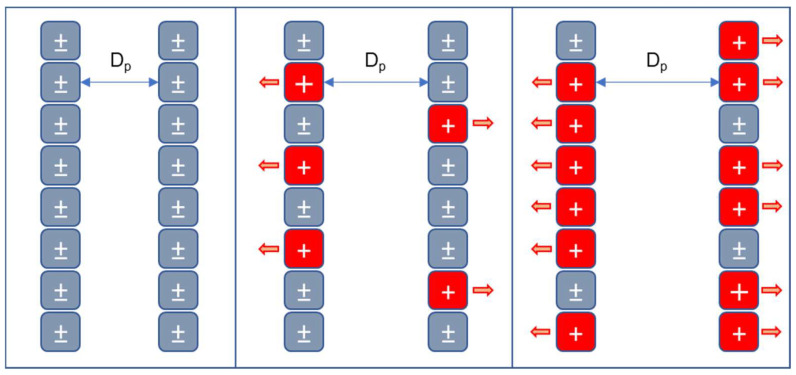
Protonation mechanism and its effect on swelling of chitosan-based cryogels.

The modification of pore geometry in the material resulting from protonation is attributed to the behavior of amino groups, this phenomenon of protonation of the pore walls as illustrated in Figure 5.

The amino groups of chitosan -NH_2_ are equivalent to neutral charges since their dipole moment is reduced; this is demonstrated by the fact that the chitosan molecule is poorly soluble in polar solutions, such as water. When protonation occurs, shown in Equation (6), these amino groups acquire positive NH_3_^+^ charges, as the number of positive charges increases, they repel each other due to the electrostatic nature of the charges, which causes the internal walls of the cryogel pores to separate, increasing the diameter of the pores, modifying their geometry, enlarging their size and improving their moisture retention capacity.

**Figure 6:**
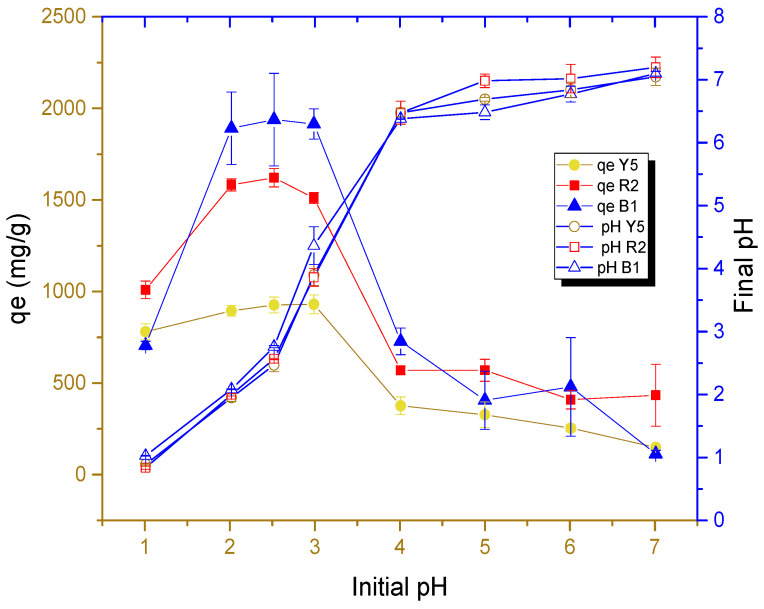
Effect of pH on the adsorption capacity of each dye at 303.15 K at a 250 mg/L concentration in solution.

Figure 6 shows the results of the effect of solution pH on the adsorption capacity of each dye in cryogel beads.

As can be seen in Figure 6, acidic media with high concentrations of hydronium ions favor the adsorption capacity of the dye on the surface of cryogels, which supports that the amino functional group protonated in acid media can be the main group involved in the adsorption of dyes. The adsorption process is primarily driven by the electrostatic attraction between the protonated groups (NH_3_^+^) and the dyes. This is because the dyes contain sulfonic functional groups (SO_3_^−^) within their chemical structure, which are negatively charged due to their high ionization in the pH range studied. This is consistent with the findings of previous experiments [14].

The adsorption capacities of each dye from the cryogel beads as a function of pH 2.5 were presented in the following order: B1 > R2 > Y5, with mean values of 1897.1, 1512.2, and 860.4 mg/g, respectively. Figure 6 shows that at a pH below 2.5, the adsorption capacity does not increase, which is explained by the fact that the -NH_2_ group bound to the glycosidic ring of chitosan reaches its ionization in this interval. This would indicate the maximum level of ionization that is proportional to the adsorption capacity and corresponds to the pKa value (2.17 to 3.18) of the conjugated amine [47], as observed in the FTIR analysis.

One of the characteristics that reinforces the zipper effect is the fact that the blue dye molecule 1 is adsorbed in higher concentrations, which is due to its low effective molecular diffusivity resulting from its high molecular weight [48]. Mass transfer mechanisms suggest that diffusivity favors the transport of the dye in solution to the active site [41]. However, analysis of the dye’s pKa through ChemAxon’s MarvinSketch software (Budapest, Hungary), version 16.7.18.0, which is part of the Marvin Beans suite as predictor software shows that the dye exhibits dipole (anionic–cationic) behavior, allowing the dye molecule to be rearranged [10]. This prevents the closure of the pores due to the zipper effect, which allows the active sites to be saturated in a greater proportion compared to red 2 and yellow 5 dyes. Another theory suggests the formation of multilayers in the adsorption of the blue dye due to this dipolar behavior [10,49].

### 2.2. Electrostatic Adsorption Mechanism

**Figure 7:**
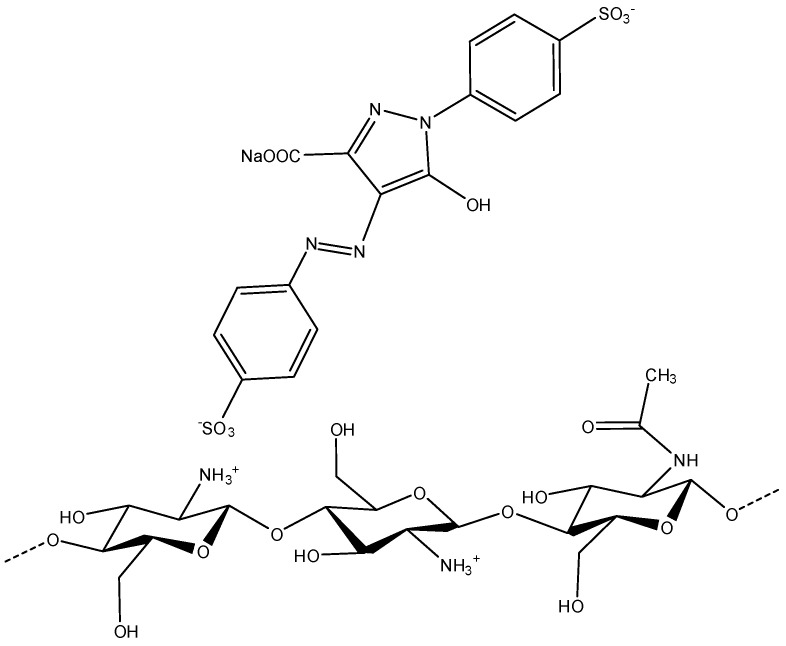
Electrostatic attraction between the cationic amino groups of chitosan and the anionic sulfonate and carboxylate groups of the dye molecule.

The adsorption process is mostly an electrostatic process, because it depends directly on the nature of the material surface, since when it is cationically bonded, it has the highest adsorption capacity. In addition to mathematical adjustments and desorption tests, this mechanism is illustrated in Figure 7.

Due to their charge difference, it is shown that these groups tend to interact, allowing them to be linked by reversible electrostatic bonds.

**Figure 8:**
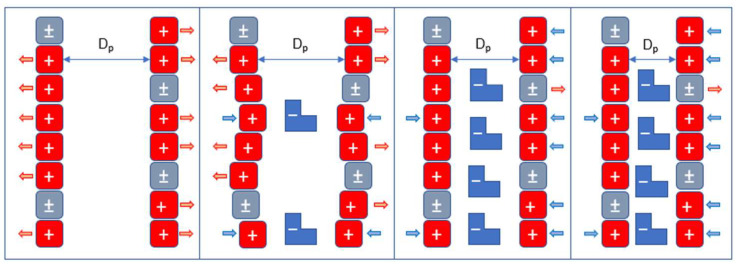
Representation of the zip closure effect caused by the physical hindrance of intraparticle diffusion in chitosan-based cryogels.

This ‘zipper’ effect (Figure 8) explains that dyes neutralize the positive charge of protonated amino groups, causing the pore walls to reduce their repulsion. This reduction allows the pore diameter to decrease, resulting in a physically reduced pore size of the cryogenic bead. The geometric change blocks and/or reduces the diffusion of dyes within the pores. The negatively charged pore walls repel the dye molecules found in the solution. Thus, the introduction of negatively charged ions leads to a decrease in both the particle diameter and water-holding capacity of chitosan-based cryogels.

The aforementioned effect was tested by measuring the moisture content of the cryogel during the adsorption process, as the cryogel beads containing absorbed dye molecules reduced in size as these molecules were adsorbed.

The moisture percentage was determined to be Y5 = 85.65%, R2 = 89.59%, and B1 = 88.44%. This indicates that the material, in the presence of dye Y5, undergoes greater physical and geometric changes in the pores. This can be explained by the neutralization of the charge of the amino group, due to the effect of the ‘zip closure’ discussed above. This limits the diffusion of the dye and, consequently, reduces the adsorption capacity of water.

It is important to note that this effect is specific to adsorbents based on biopolymers such as chitosan. The elasticity of polymers and crosslinkers is the main cause of the change in pore geometry, as well as the presence in large quantities of protonated hydronium ions and amino groups. According to tests and comparisons made with other materials containing cellulose and polyvinyl alcohol, the cryogel preparation methodology is what allows a homogeneous distribution of the polysaccharide and crosslinker [20].

**Figure 9:**
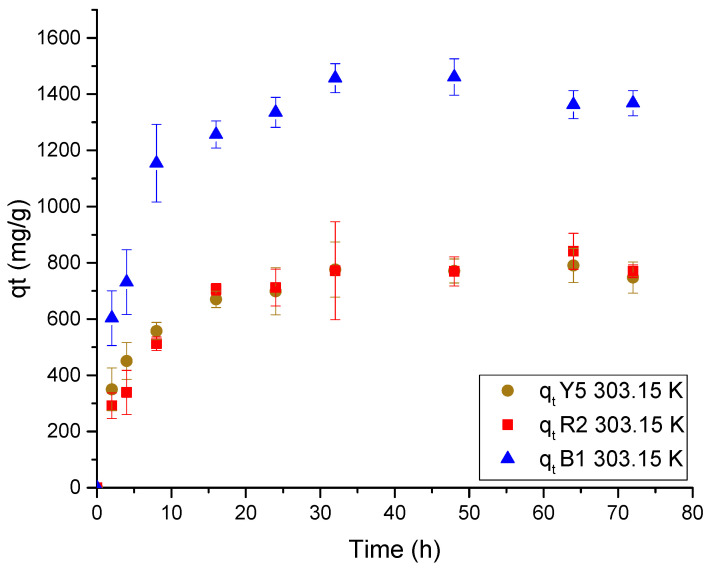
Adsorption kinetic data for each dye in Ch-C-EGDE cryogel at 303.15 K.

Figure 9 shows the results of the adsorption kinetics of the dyes, illustrating the impact of the change in molecular geometry and its effect on the size and moisture percentage of the cryogel.

The equilibrium for adsorption of the three dyes is reached between 16 and 24 h. However, after 8 h, the adsorption rate decreases due to the amount of dye retained in the pores of the cryogel, which is related to the ‘zipper effect’. This effect causes the adsorption to behave similarly to a chemical reaction, where the reaction rate slows as the chemical reactants are consumed [14,50,51,52].

On the other hand, upon analyzing the morphology and effect of the protonated media, it is observed that the adsorption rate decreases when the amino sites are occupied by the dyes. This is due to a reduction in the availability and accessibility of the protonated sites caused by the adsorption of anionic dye molecules. These molecules neutralize the cationic charges of the protonated amino group and diffuse through the pores of the cryogel, repelling similar loads.

The data fit better to the Ho model, which represents chemisorption, as shown in Table 3. This model assumes that the adsorption rate decreases as the active sites in the pores are occupied. It interacts with the dyes in solution, limiting the diffusion of trapped particles, which is well explained by the zipper closure effect. The constant K_2_ of the Ho model suggests that the interaction of the dyes under the same conditions varies for each dye. This implies that the constant is related to the molecular diffusivity coefficient (m^2^/s) of each dye, where values of D_Y5_ = 3.81 × 10^−10^ m^2^/s, D_R2_ = 3.48 × 10^−10^ m^2^/s, and D_B1_ = 2.89 × 10^−9^ m^2^/s were obtained using the Wilke–Chang equation (Equation (7)) [48]. However, the relationship between the two is not clearly observed, as the blue dye 1 has a low molecular diffusion coefficient.

(2) $$D_{AB}=\frac{8.029\times 10^{-16}\;T}{\mu V_{A}^{3/5}}$$

where $D_{AB}$ is the molecular diffusion coefficient (m^2^/s) of an element in liquid water, T is the temperature of the solution in K, μ is the viscosity of water in Pa s, and $V_{A}$ is the molar volume in m^3^/kmol.

**Figure 10:**
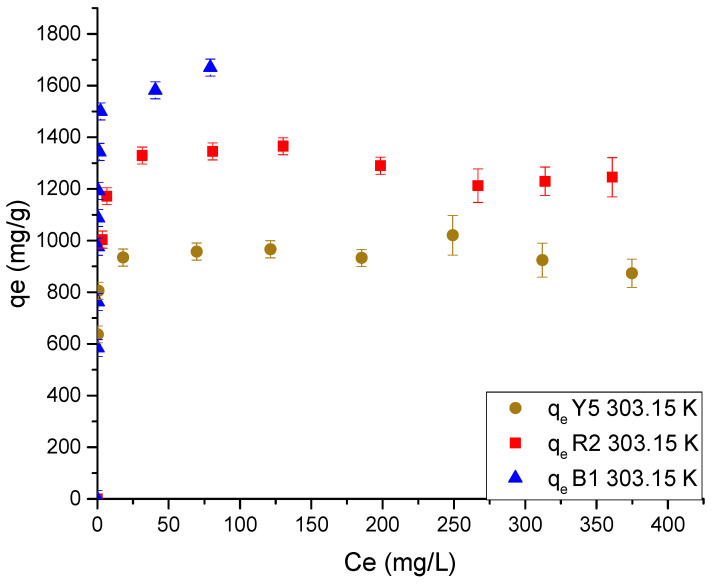
Data of the adsorption isotherm of each dye in Ch-C-EGDE cryogel at 303.15 K.

Additionally, the effect of adsorption equilibrium on pore geometry was analyzed by comparing the adsorption capacity. The results of this analysis are presented in Figure 10.

Figure 10 shows a trend in adsorption capacity similar to that observed in adsorption kinetics. The same adsorption hierarchy of B1 > R2 > Y5 was presented, with equilibrium capacity values of 1605.45, 1278.90, and 945.06 mg/g, respectively.

The explanation for this can be attributed to the speciation of the dyes and their molecular diffusivity coefficient. Red 2 and yellow 5 are anionic dyes, while blue 1 is a dipolar dye that prevents zipper closure [10]. The dyes red 2 and yellow 5 are dominated by the diffusion coefficient, which implies that the rate at which the anionic dyes are adsorbed is proportional to the activation of the zipper closure, which is responsible for the greater interference with the diffusion of the dye molecules as the dyes are sorbed [51]. This effect, in addition to modifying the adsorption rate, also has an implication in the availability and saturation of the amino proton groups, due to the reduction in pore size, which limits the dye molecules from reaching the active sites [51].

**Figure 11:**
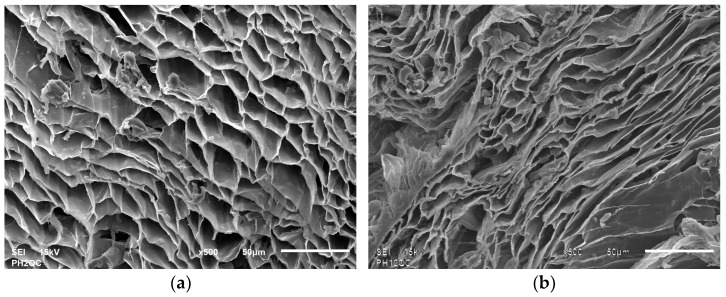
Comparison of cryogel morphology at pH 2 (**a**) and pH 12 (**b**) at 15 kV and 500×.

This limited accessibility to the active sites of the material has been corroborated using SEM analysis of cryogel under acidic and alkaline conditions, where pores change from 4–12 μm at pH 2 to 2–6 μm at pH 12 (refer to Figure 11).

From the results of the fitting of the data to the mathematical models of adsorption equilibrium shown in Table 4, it is observed that the data fit better to the Sips isotherm, where the value of the coefficient *n* is similar to the Langmuir isotherm.

The mathematical adjustment allows us to assume that the adsorbate reaches the active sites, generating a monolayer of homogeneous form and using more than one active site per dye molecule, characteristic of chemisorption. However, the adsorption mechanism is determined by the electrostatic forces of the protonated amino groups and the anionic molecules. This indicates that the zipper closure effect distributes the dye heterogeneously in the protonated functional groups, leaving gaps in the material due to the geometric impediment on the diffusivity of the dye [53,54].

Table 5 allows us to compare the results of the adsorption capacity obtained in this study with those obtained from similar studies of dye adsorption in scientific literature.

From the data in the table above, the adsorption capacity is greater than that of other dyes, mainly due to the fact that the selection of the dye favors adsorption, that the dyes used in this study are cationic in nature, and that, therefore, they require a modification of the solution pH to achieve their maximum adsorption capacity [14].

### 2.3. Desorption Process Results

The results of the desorption test are shown below, which help to demonstrate the reversible adsorption process.

**Figure 12:**
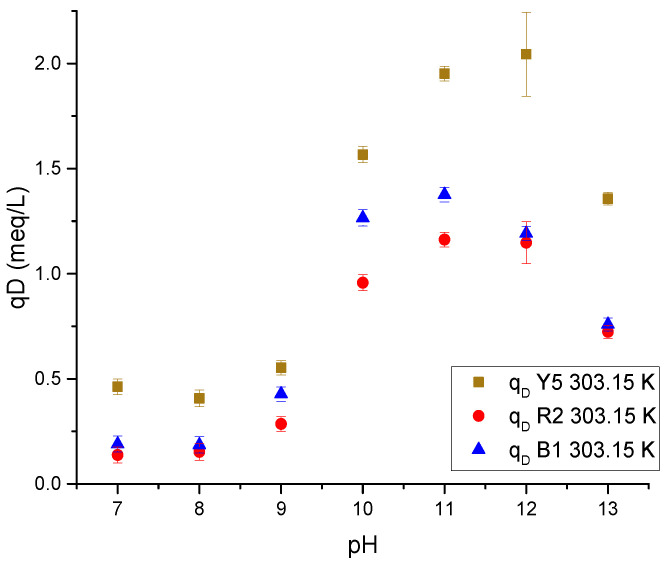
The pH influence on dye desorption in a single-component Ch-C-EGDE cryogel system.

Figure 12 shows the effect of increasing pH on cryogel. In the alkaline ranges, the presence of hydroxyl ions is favored, which reduces the ionization of the amino groups and neutralizes the NH^3+^ charges. On the other hand, the hydroxyl and carbonyl groups of chitosan and cellulose, respectively, are ionized with a negative charge, repelling the anionic dyes.

### 2.4. Desorption Kinetics

**Figure 13:**
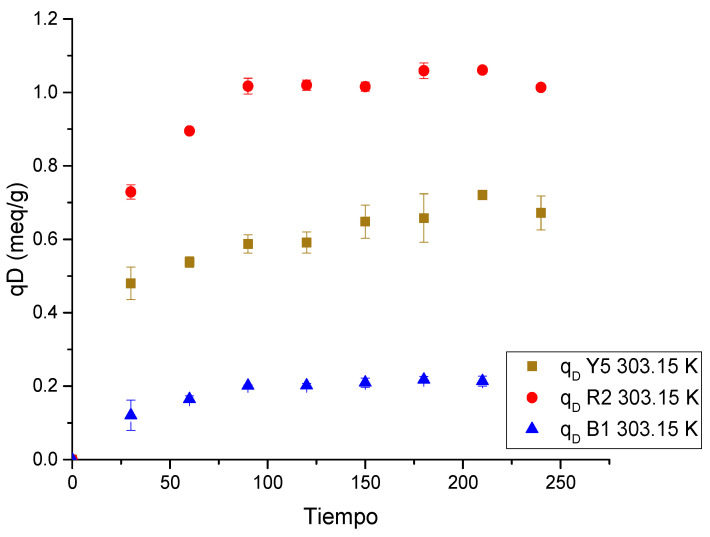
Dye desorption kinetics in a single-component Ch-C-EGDE cryogel system.

Desorption kinetics (Figure 13) show the time to achieve charge equilibrium in solution and on the surface of the chitosan-based cryogels. The pH was preserved during the test in a range of 11.12 ± 0.23.

The desorption process of the dyes is very fast across all three replicate experiments, since equilibrium is reached at 150 min for the three dyes with Ch-C-EGDE cryogel beads, demonstrating that desorption is much faster than adsorption.

On the other hand, when comparing the ionization rate at pH 12, it can be observed that the time is similar; therefore, the desorption rate has a direct relationship with the ionization rate of chitosan cryogels.

As the functional groups are deprotonated, the number of negative charges within the cryogel pores increases, repelling the dye anions previously adsorbed on the surface of the chitosan-based cryogels. However, the effect of pH blocks the pores, thus limiting the desorption rate.

In the case of red 2 dye, Ch-C-EGDE cryogel has an adsorption capacity of up to 699 mg/L. During the first and second stages of the regeneration cycles, the material loses 45% of its adsorption capacity, which is mainly due to the zip effect, as the closure of the pores also limits the dyes’ adsorption.

**Figure 14:**
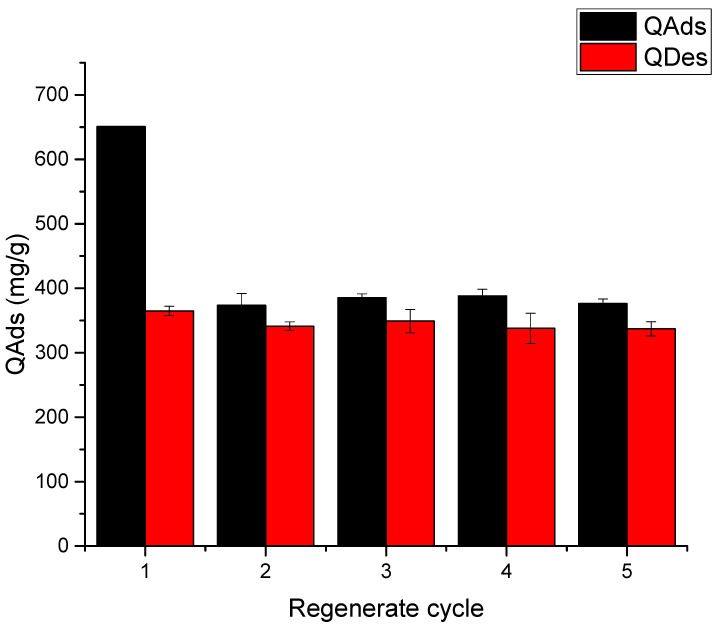
Tests of the Ch-C-EGDE cryogel reuse cycle for dye FD&C yellow 5.

As shown in Figure 14, Ch-C-EGDE cryogel beads exhibit a 42% decrease in adsorption capacity between the first and second cycle for all three dyes at pH 12, concomitant with until 60% reduction in bead diameter post-desorption. This confirms the ‘zipper effect,’ where pore collapse under alkaline conditions permanently traps dye molecules in the cryogel matrix, limiting full regeneration.

## 3. Conclusions

This study demonstrates that dye adsorption onto chitosan–cellulose cryogel beads (Ch-C-EGDE) is governed by electrostatic interactions between the protonated amino groups (−NH_3_^+^) and anionic functional groups (−SO_3_^−^) of the dyes, with a high dependence on solution pH. Acidic media (pH 2.5) promote protonation, enhance adsorption performance, and expand pore structures due to electrostatic repulsion within the polymer matrix.

The Ch-C-EGDE cryogel beads undergo reversible morphological changes, most notably in pore size and overall bead geometry during adsorption and desorption. Under acidic conditions, bead diameter increases by up to 80%, whereas under alkaline conditions, it contracts by as much as 60%. This “zipper effect,” caused by protonation and subsequent dye binding at the pore walls, directly modulates dye diffusion and active-site accessibility. Differences in dye speciation and molecular diffusivity further account for the adsorption hierarchy (B1 > R2 > Y5), with maximum capacities of 1505.6, 869.8, and 808.4 mg g^−1^, respectively—values that surpass those reported for similarly sized chitosan-based adsorbents.

Kinetic analysis shows the pseudo-second-order (PSO) model provides the best fit, while equilibrium data conform to the Sips isotherm, indicating heterogeneous, multilayer chemisorption governed by specific dye–adsorbent interactions. Compared to other chitosan-derived materials, the Ch-C-EGDE cryogel offers superior adsorption performance, underscoring its promise as an efficient and sustainable adsorbent for dye-laden wastewater. Its rapid desorption kinetics and retention of structural integrity over multiple cycles further highlight the material’s excellent reusability and stability.

## 4. Materials and Methods

### 4.1. Reagents and Materials

The solutions were prepared using the following reagents and materials: industrial-grade chitosan (Ch) from Alimentos America (Guadalajara, Mexico; lot K1202029; 84.54% degree of deacetylation, Mv = 82 682.5 g·mol^−1^); microgranular cellulose (C) from Sigma–Aldrich (St. Louis, MO, USA); and ethylene glycol diglycidyl ether (EGDE) from Tokyo Chemical Industry Co. (Tokyo, Japan). FD&C Yellow 5 (Y5; CI 19140), FD&C Red 2 (R2; CI 16185), and FD&C Blue 1 (B1; CI 42090; 85% food grade) were purchased from Frallier (aceites y escencias s.a.) (Mexico City, Mexico). Glacial acetic acid, hydrochloric acid, and sodium hydroxide (analytical grade) were supplied by Fermont (Monterrey, Mexico). Purified water was obtained using an Arium Pro water purification system (Sartorius, Göttingen, Germany).

### 4.2. Synthesis of Chitosan-Based Cryogel

**Figure 15:**
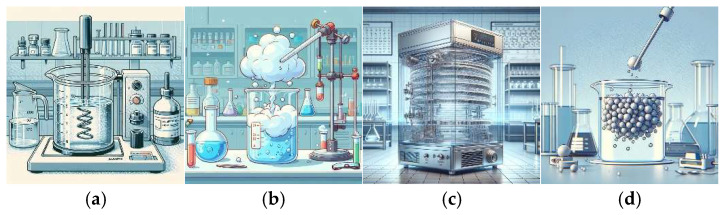
Diagram of the synthesis of cryogel Ch-C-EGDE: (**a**) Preparation of gel with chitosan and acetic acid 0.4 M. Addition of cellulose in a ratio of 1.9:1. Binding with EGDE covalent crosslinker. (**b**) Dripping in liquid air. (**c**) Freeze-drying process at 0.008 mbar and 188 K for 24 h. (**d**) Cryogel washing in distilled water until the pH of the water is neutral.

The Ch-C-EGDE gel was prepared by dissolving 3.8 g of chitosan in 100 mL of 0.4 M acetic acid under moderate stirring on a Thermo Scientific S194615 Cimarec analog magnetic stirrer (ceramic platform 4.25″ × 4.25″) using a 2″ PTFE-coated stir bar. Stirring was set to a variable speed (100–1500 rpm) to compensate for increasing viscosity. Complete chitosan dissolution required approximately 2 h. Then, 2 g of microgranular cellulose was added, and the suspension was stirred under the same conditions for an additional 30 min to ensure full dispersion. Finally, ethylene glycol diglycidyl ether (0.1% *w*/*w* of chitosan) was introduced, and the mixture was stirred for 45 min at room temperature to achieve a homogeneous gel prior to cryogelation (see Figure 15a) [14].

The synthesized gel was added by drip to liquid air (see Figure 15b), and the frozen beads formed were freeze-dried in a Labconco FreeZone freezing-drier at 0.008 mbar and 188 K for 24 h (see Figure 15c). The resulting dried beads were washed with distilled water and stored for later use (see Figure 15d).

### 4.3. Material Characterizations

The moisture content of the synthesized Ch-C-EGDE cryogel beads was determined using the gravimetric method (Equation (1)). Samples were dried in a desiccator over silica gel at room temperature and weighed every 24 h until the results of consecutive measurements differed by less than 0.1%. All determinations were carried out in triplicate at pH 6.0 and across the pH range 2–11 using a Mettler Toledo (Greifensee, Switzerland) New Classic MF analytical balance (model ML204/03).

(3) $$\%M=\frac{W_{W}-W_{D}}{W_{W}}\times 100$$

where %*M* is the moisture content, *W_W_* is the mass of the cryogel before drying, and *W_D_* is the mass after drying.

Three representative pH values: 2.5, 6.0, and 12.0 were selected to investigate the pH-dependent swelling behavior of cryogel beads. The acidic condition (pH 2.5) induces extensive protonation of chitosan’s amino groups, leading to increased electrostatic repulsion and expansion of the polymer network. The near-neutral condition (pH 6.0) reflects environmentally and physiologically relevant settings, offering practical applicability. In contrast, the alkaline condition (pH 12.0) promotes deprotonation of amino groups, thereby reducing charge density and decreasing swelling capacity. In each case, 50 mg of Ch-C-EGDE beads were immersed in 50 mL of the corresponding buffer at 298 K under gentle stirring (100 rpm). Beads were removed at predetermined time intervals (5, 15, 30, 60, 90, 120, and 150 min), blotted to remove surface liquid, and weighed to calculate moisture retention.

Scanning electron microscopy (SEM) was used to determine the cryogel’s morphological characteristics. A Jeol equipment model JSM-6610LV was used, applying a voltage of 20 kV and with magnifications ranging from ×30 to ×1000. The adsorbent functional groups were determined using the FTIR technique, with an Agilent (Santa Clara, CA, USA) Varian 640-IR model, in a range from 4000 to 550 cm^−1^, with 16 scans per second, at a resolution of 4 cm^−1^ and with ATR coupling.

The specific surface area was determined by adjusting the nitrogen physisorption data and its hysteresis cycle through the Brunauer–Emmet–Teller equation, using BELSORP-Max equipment (Osaka, Japan) and BELMaster software (version 6.3). A pretreatment was applied at 343 K and 10^−2^ Pa in the VelPrep_VACII_ equipment.

### 4.4. pH Point of Zero Charge

The point of zero charge (pHpzc) was determined using the salt addition method [61]. In each experiment, 0.25 g of cryogel was added to 20 mL of 0.1 M NaCl solution; the initial pH (pH_i_) was adjusted from 1 to 13 (±0.05 units) using 0.1 M HCl or NaOH and recorded with a Thermo Fisher (Waltham, MA, USA) pH meter. Samples were stirred in a Heidolph (Schwabach, Germany) Unimax 1010 orbital shaker at 303.15 K and 200 rpm for 24 h. After equilibration, the supernatant was separated and the final pH (pH_f_) measured. For each pH_i_, the difference ΔpH = pH_f_ − pH_i_ was calculated and plotted against pH_i_; the pH_pzc_ was taken as the pH_i_ value at which ΔpH = 0. All measurements were carried out in triplicate.

### 4.5. Adsorption Experiments

The powdered dyes were dissolved in distilled water to the required concentrations. The pH was adjusted using HCl and/or NaOH solutions. The experiments were conducted using a ratio of 0.24 g of cryogel (dry mass) per liter of dye solution at a concentration of 250 mg/L. These conditions were selected based on previous optimization work reported by García-González et al. (2021), where the same bead-to-dye ratio and contact time yielded 70–80% removal efficiency at equilibrium [14]. The experiments were performed in triplicate at 200 rpm and 303.15 K temperature with a heating and shaking dry bath (Heidolph Unimax 1010). The concentration of each dye was measured at the start and end of each experiment using a Thermo (Waltham, MA, USA) Genesys 10S UV–Vis Spectrophotometer at the maximum absorbance wavelengths for each dye (Y5 at 426 nm, R2 at 520 nm, and B1 at 626 nm).

The adsorption capacity of the material (mg/g) was calculated using Equation (2).

(4) $$q=((C_{i}-C_{f})\;V)/M$$

where the amount of dye adsorbed per unit mass of the adsorbent at equilibrium (mg/L) is represented by q. C_i_ represents the initial concentration of the solute in the solution in mg/L, while C_f_ represents the concentration of the solute at a given follow-up time of the adsorption process in mg/L. V represents the used volume of the solution containing the solute in L, and M represents the mass of the adsorbent in g.

The effect of the pH of the solution on the adsorption capacity for each dye was evaluated. For this purpose, cryogel Ch-C-EGDE was added to solutions with an initial concentration of 250 mg/L of each dye at different pH values (ranging from 1 to 7), for 72 h at a temperature of 303.15 K.

In order to perform the kinetic adsorption test, the adsorption capacity of the cryogel was determined at different times (0.5 to 72 h) at 303.15 K and at a concentration of 250 mg/L for each dye. Fixed amounts of adsorbent (0.24 g/L) were used at each of the previously selected pHs.

The kinetic models used in this article are described below:

(5) $$q_{t}=q_{e}(1-e^{-k_{1}t})\;\mathrm{Pseudo-first}\;\mathrm{order}\;\mathrm{equation}\;(\mathrm{PFO})$$

(6) $$q_{t}=\frac{tk_{2}q_{e}^{2}}{1+k_{2}q_{e}t}\;\;\mathrm{Pseudo-second}\;\mathrm{order}\;\mathrm{equation}\;(\mathrm{PSO})$$

(7) $$q_{t}=\frac{1}{\beta }\;\ln(1+\alpha \beta t)\;\mathrm{Elovich}\;\mathrm{equation}$$

where k_1_: s^−1^, k_2_: g mg^−1^ s^−1^ pseudo adsorption rate constant; q_e_ maximum amount of adsorbate sorbed at equilibrium (mg g^−1^); q_t_ is the adsorption capacities at time t, t is time, α is the initial sorption rate in the Elovich model (mg g^−1^ s^−1^); β is the constant related to the extent of surface coverage and activation energy for chemisorption in the Elovich model (g mg^−1^).

Equilibrium adsorption data were fitted to three widely used isotherm models:

(8) $$q_{e}=\frac{q_{m}\;K_{L}\;C_{e}}{1+K_{L}C_{e}}\;\;\;\mathrm{Langmuir}\;\mathrm{isotherm}$$

(9) $$q_{e}=K_{F}\;{C_{e}}^{1/n}\;\;\;\;\mathrm{Freundlich}\;\mathrm{isotherm}$$

(10) qe=qmKS Ce1ns1+KS Ce1ns   Sips isotherm

where $C_{e}$ is the concentration of dye at equilibrium (mg/L), $q_{e}$ is the amount of dye adsorbed at equilibrium (mg/g); $q_{m}$ is the maximum capacity at equilibrium (mg/g), $K_{L}$ is the constant of Langmuir (L/mg), which indicates the binding force between the dye molecules and the chitosan. $K_{F}$ represents the Freundlich constant (mg/g), and *n* is considered the heterogeneity of the adsorbent surface and its affinity for the adsorbent. $K_{S}$ is the count of Sips (L/g); $n_{s}$ the exponent of Sips, if $n_{s}$ is equal to the value of 1, the equation of Sips is reduced to the equation of Langmuir, which is applied to ideal surfaces

Adsorption equilibrium studies were conducted to determine the adsorption capacity of the cryogel using fixed amounts of 0.24 g/L of adsorbent and different initial concentrations in solution for each dye (between 150 and 450 mg/L). The samples were left in contact for 48 h, at a pH of 2.5 in equilibrium that favored adsorption, which was determined based on optimal values from previous tests.

Although originally developed for gas-phase systems, the Langmuir, Freundlich, and Sips isotherms have been widely adapted to describe solute adsorption on polymeric and cryogel surfaces. The Langmuir model assumes monolayer adsorption onto a homogeneous surface with finite, energetically equivalent active sites, and its shape reflects linear uptake influenced primarily by physical or molecular diffusion processes. In contrast, the Freundlich isotherm accounts for heterogeneous surface energies and multilayer formation, providing flexibility to describe systems where adsorption is influenced by surface heterogeneity and solute–solute interactions. The Sips isotherm combines both models; it behaves like Freundlich at low solute concentrations and converges toward Langmuir behavior at high concentrations, making it especially suitable for systems like chitosan–cellulose cryogels where pore distribution and surface chemistry change with pH and dye loading.

### 4.6. Desorption Process

#### 4.6.1. The pH Effect on Desorption

The pH effect on the desorption capacity of Ch-C-EGDE cryogel was evaluated by preparing alkaline solutions and using NaOH adjusted to pH 8, 9, 10, 11, and 12. For each condition, 24 mg of dye-saturated cryogel beads were immersed in 50 mL of the solution for 24 h at room temperature. The supernatant was analyzed to determine the desorption capacity (q_D_) using Equation (9), and the final pH was recorded to confirm equilibrium conditions.

(11) $$q_{D}=\frac{C_{D}\;V}{m}\;\;\mathrm{Desorption}\;\mathrm{capacity}\;\mathrm{equation}$$

where *q_D_* is desorption capacity (*q_D_*, mg/g), *C_D_* (mg/L) is the dye concentration in the desorption solution at equilibrium (or at time t), *V*(L) is the volume of the desorption solution, and *m* (g) is the dry mass of cryogel beads used.

#### 4.6.2. Desorption Kinetics

After selecting the optimal pH (11), continuous desorption was performed for 4 h by immersing cryogel beads (previously saturated with individual dyes) into an alkaline solution adjusted to pH 11 using NaOH. The desorbing solution volume was set to 1/10 of the original adsorption volume. Throughout the desorption process, the supernatant was periodically sampled and analyzed for residual dye concentration, while pH was maintained through regular adjustment. Notably, changes in dye tonality under alkaline conditions were attributed to speciation phenomena, consistent with previous characterizations based on the pKa profiles of each dye.

At the end of desorption, cryogels were washed with distilled water until the wash water pH equaled that of the distilled water, followed by a final rinse with pH 2.5 water. The cryogels were re-saturated in a dye solution at 250 mg/L and pH 2.5 for 48 h, repeating the adsorption–desorption cycle five times.

### 4.7. Data Analysis

All kinetic and equilibrium data were modeled using the equations presented above. Swelling and adsorption kinetics were fitted to the pseudo-first-order, pseudo-second-order, and Elovich models, while equilibrium adsorption data were fitted to the Langmuir, Freundlich, and Sips isotherms. Non-linear least-squares regressions were performed in OriginPro 2016 (OriginLab Corp.) using the Levenberg–Marquardt algorithm. Goodness-of-fit was evaluated using the correlation coefficient (R^2^) and the sum of squared errors.

## Figures and Tables

**Table 1:** Specific surface area results obtained for cryogel beads.

Parameter	Units	Quantity
S_a,BET_	m^2^/g	10.22
Mean Pore volume	cm^3^/g	0.013
Mean Diameter pore	nm	4.96

**Table 2:** Fitting swelling kinetics data to mathematical models.

pH	PFO	PSO
2.5	q_e_ = 0.94528 mg g^−1^	q_e_ = 0.9466 mg g^−1^
	K_1_ = 0.183 min^−1^	K_2_ = 5.26 g mg^−1^ min^−1^
	R^2^ = 0.9999 χ^2^ = 6.37 × 10^−6^	R^2^ = 0.9999 χ^2^ = 4.23 × 10^−6^
6	q_e_ = 0.91725 mg g^−1^	q_e_ = 0.91706 mg g^−1^
	K_1_ = 0.209 min^−1^	K_2_ = 1.48 × 10^44^ g mg^−1^ min^−1^
	R^2^ = 0.9995 χ^2^ = 4.38 × 10^−5^	R^2^ = 0.9995 χ^2^ = 4.41 × 10^−5^
12	q_e_ = 0.90963 mg g^−1^	q_e_ = 0.91175 mg g^−1^
	K_1_ = 0.192 min^−1^	K_2_ = 3.789 g mg^−1^ min^−1^
	R^2^ = 0.9997 χ^2^ = 2.02 × 10^−5^	R^2^ = 0.9998 χ^2^ = 1.38 × 10^−5^

**Table 3:** Summary of data fitting parameters to kinetic models.

	PSO	PFO	Elovich
Y5	q_e_ = 808.42 mg g^−1^	q_e_ = 742.89 mg g^−1^	α = 1352.06 mg g^−1^ min^−1^
	K_2_ = 4.05 × 10^−4^ g mg^−1^ min^−1^	K_1_ = 0.227 min^−1^	β = 8.17 × 10^−3^
	R^2^ = 0.9916	R^2^ = 0.9667	R^2^ = 0.9808
R2	q_e_ = 869.81 mg g^−1^	q_e_ = 777.77 mg g^−1^	α = 517.71 mg g^−1^ min^−1^
	K_2_ = 2.29 × 10^−4^ g mg^−1^ min^−1^	K_1_ = 0.152 min^−1^	β = 6.42 × 10^−3^
	R^2^ = 0.9850	R^2^ = 0.9777	R^2^ = 0.9672
B1	q_e_ = 1505.64 mg g^−1^	q_e_ = 1383.88 mg g^−1^	α = 2264.10 mg g^−1^ min^−1^
	K_2_ = 2.08 × 10^−4^ g mg^−1^ min^−1^	K_1_ = 0.218 min^−1^	β = 4.33 × 10^−3^
	R^2^ = 0.9812	R^2^ = 0.9807	R^2^ = 0.9462

**Table 4:** Summary of the parameters of fitting the data to the isotherm models.

	Freundlich	Langmuir	Sips
Y5	K_F_ = 769.21 mg g^−1^ (mgL^−1^)^−1/n^	q_max_ = 946.91 mg g^−1^	q_max_ = 945.06 mg g^−1^
	*n* = 24.98	K_L_ = 6.71 L mg^−1^	K_S_ = 5.78
	R^2^ = 0.6512	R^2^ = 0.8802	*n* = 1.18
			R^2^ = 0.8830
R2	K_F_ = 1092.82 mg g^−1^ (mgL^−1^)^−1/n^	q_max_ = 1298.40 mg g^−1^	q_max_ = 1287.90 mg g^−1^
	*n* = 33.27	K_L_ = 1.12 L mg^−1^	K_S_ = 0.55
	R^2^ = 0.9560	R^2^ = 0.9806	*n* = 1.87
			R^2^ = 0.9847
B1	K_F_ = 1103.86 mg g^−1^ (mgL^−1^)^−1/n^	q_max_ = 1658.21 mg g^−1^	q_max_ = 1605.70 mg g^−1^
	*n* = 9.41	K_L_ = 3.34 L mg^−1^	K_S_ = 3.32
	R^2^ = 0.8590	R^2^ = 0.9799	*n* = 1.35
			R^2^ = 0.9858

**Table 5:** Comparison of the adsorption capacity obtained in this study and other dye adsorption capacities obtained from the scientific literature.

Reference	Dye	q_m_ (mg/g) Chitosan-Based Adsorbents
Doondani et al., 2024 [55]	RB 19, RO 16, CR	219.6, 129.6, 118.8
Gomase et al., 2024 [56]	RB19	323.15
Doondani et al., 2022 [57]	RR	125.1
Nandanwar et al., 2022 [58]	RBBR	540.3
Nandanwar et al., 2023 [59]	RO	34.61
El Kaim Billah et al., 2024 [60]	MO	94.8
This study	Y5, R2, B1	808.4, 869.8, 1505.6

## Data Availability

The data supporting the results of this study are available from the corresponding author upon reasonable request. Due to potential ethical or confidentiality restrictions, the data are not in a public repository but can be shared under appropriate terms agreed upon with the parties involved.

## Abbreviations

The following abbreviations are used in this manuscript:

PSO	Pseudo Second Order
PFO	Pseudo First Order
Ch-C-EGDE	Beads of chitosan–cellulose and ethylene glycol diglycidyl ether
Y5	FD&C Yellow 5, Dye alimentary
